# Gynaecological cancers and their cell lines

**DOI:** 10.1111/jcmm.16397

**Published:** 2021-03-02

**Authors:** Kristijan Skok, Lidija Gradišnik, Uroš Maver, Nejc Kozar, Monika Sobočan, Iztok Takač, Darja Arko, Rajko Kavalar

**Affiliations:** ^1^ Department of pathology General Hospital Graz II Graz Austria; ^2^ Institute of Biomedical Sciences Faculty of Medicine University of Maribor Maribor Slovenia; ^3^ Division of Gynecology and Perinatology University Medical Center Maribor Maribor Slovenia; ^4^ Faculty of Medicine University of Maribor Maribor Slovenia; ^5^ Department of Pathology University Medical Center Maribor Maribor Slovenia

**Keywords:** breast neoplasm, cell line, cervix cancer, endometrial neoplasms, gynaecology, in vitro techniques, pathology, tumour cell line

## Abstract

Cell lines are widely used for various research purposes including cancer and drug research. Recently, there have been studies that pointed to discrepancies in the literature and usage of cell lines. That is why we have prepared a comprehensive overview of the most common gynaecological cancer cell lines, their literature, a list of currently available cell lines, and new findings compared with the original studies. A literature review was conducted via MEDLINE, PubMed and ScienceDirect for reviews in the last 5 years to identify research and other studies related to gynaecological cancer cell lines. We present an overview of the current literature with reference to the original studies and pointed to certain inconsistencies in the literature. The adherence to culturing rulesets and the international guidelines helps in minimizing replication failure between institutions. Evidence from the latest research suggests that despite certain drawbacks, variations of cancer cell lines can also be useful in regard to a more diverse genomic landscape.

## 1 INTRODUCTION

**FIGURE 1:**
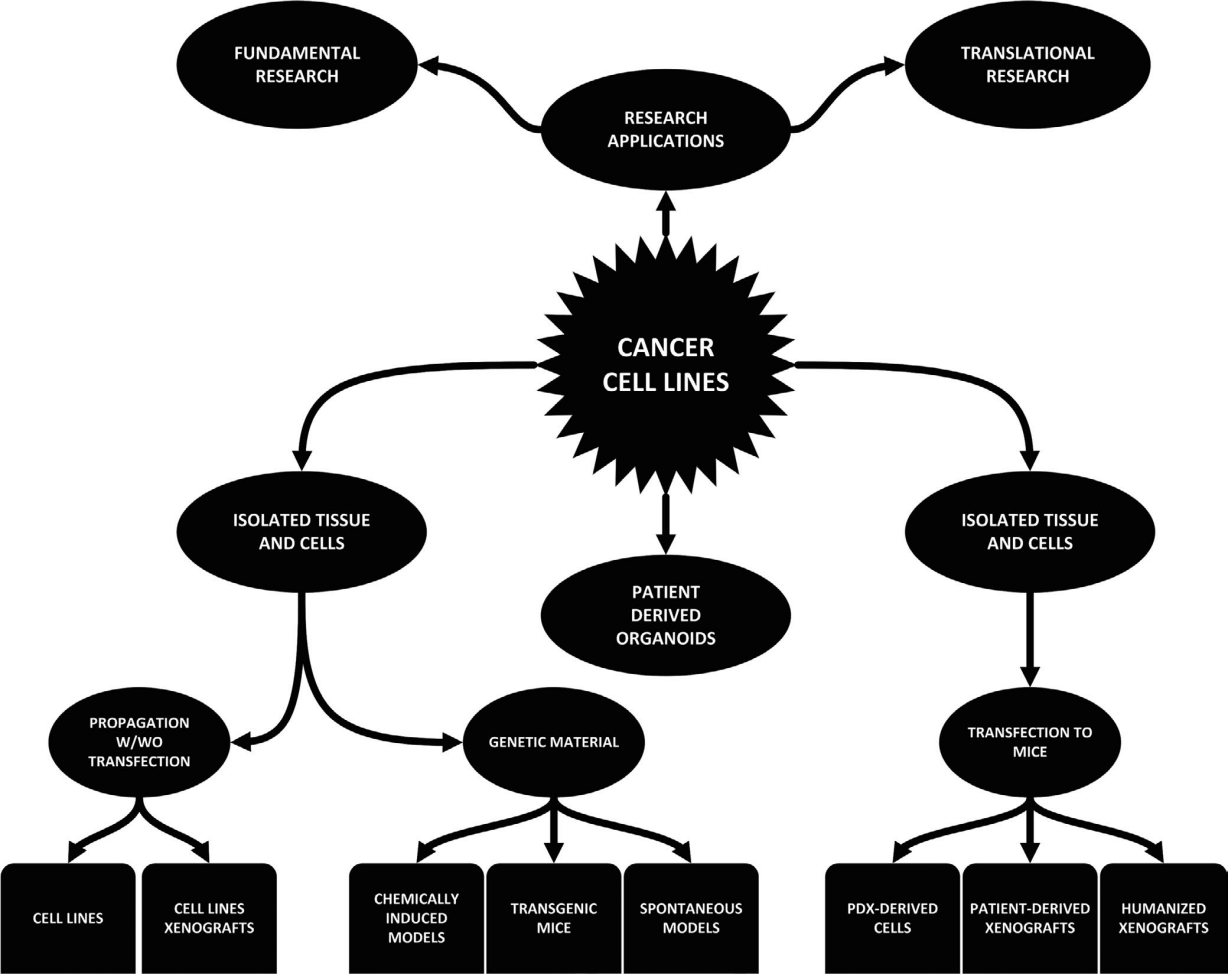
Potential CCL applications in research

There is a variety of methods to study the properties of malignancies. However, many of those methods depend on clinical subjects and/or patients and are in certain regards restricted/limited. A possible to avenue to form a better understanding of the carcinogenesis and behaviour of specific malignancies are cell cultures and cell lines (CLs).^1^, ^2^, ^3^ Areas that commonly utilize CLs are pathology and oncology as well as pre‐clinical areas such as pharmacology. Research on cancer cell lines (CCLs) in these areas holds the potential to lead to translational, clinically applicable results. Several papers have discussed and compared different cell models. This includes CLs, in vivo models, or cell models that are derived from individual patients. It comes as no surprise that all have their drawbacks and advantages. One of the oldest and still most recurring problems in CL culturing remains to be the potential of genetic or epigenetic changes can potentially arise during their growth and use. The latter limits the correlation potential of results based on these with the properties of the primary tissue.^2^, ^4^, ^5^ By this, also their clinical relevance might be questioned. Nevertheless, there are several obvious benefits of CCLs. Figure 1 shows some of these potential applications of CCLs in medicine and translational research.

The limitations of CCLs, considerations about their relevance for clinical outcomes, and the discrepancies in their characteristics through the decades, were discussed before. For example, a recent paper by Ben‐David et al presented evidence that points to a variety of mutational processes that affect the homogeneity of commonly used CCLs. On a practical level, this translates to the phenotypical characteristics of these CLs and their behaviour (therapy response).^6^, ^7^ Consequently, when considering the replicability of experiments, one understands that a number of factors can alter the final outcome. Some of these are as follows: (a) the number of population doublings that affect the cell geno‐ and phenotypes; (b) the origin of CCLs could be from the primary source tissue or a metastasis; (c) culture conditions could promote differentiation or even dedifferentiation. Despite this seemingly ‘negative’ prognosis to CL use, multiple mechanisms and rules have been proposed that help either prevent or at least help resolve these and other occurrences. Some of these recommendations and guidelines will be discussed in the later sections of the review.

In comparison with complex culturing techniques (eg transgenic mice, transfection‐based models and xenografts), CLs still provide a combination of a stable culturing setting, control over the experiment, relatively quick results and moderate expenses.^8^, ^9^ Furthermore, thanks to recent advances in genomic studies and the digitalization of data, specific CL properties can be simply checked via various databanks, including their possible uses and genetic heritage (eg Cellosaurus, ECLA).^10^, ^11^, ^12^, ^13^

We present an overview of three types of gynaecological CCLs. Firstly, we present our methodology of search. The next chapter will discuss breast cancer (BC) and its CLs, the following endometrial cancer (EC) and its CLs, the subsequent cervical cancer (CC), and its CLs. Finally, the last chapter will be dedicated to new emerging and potential methods of use for CL research and our own related experiences.

## 2 METHODS

**FIGURE 2:**
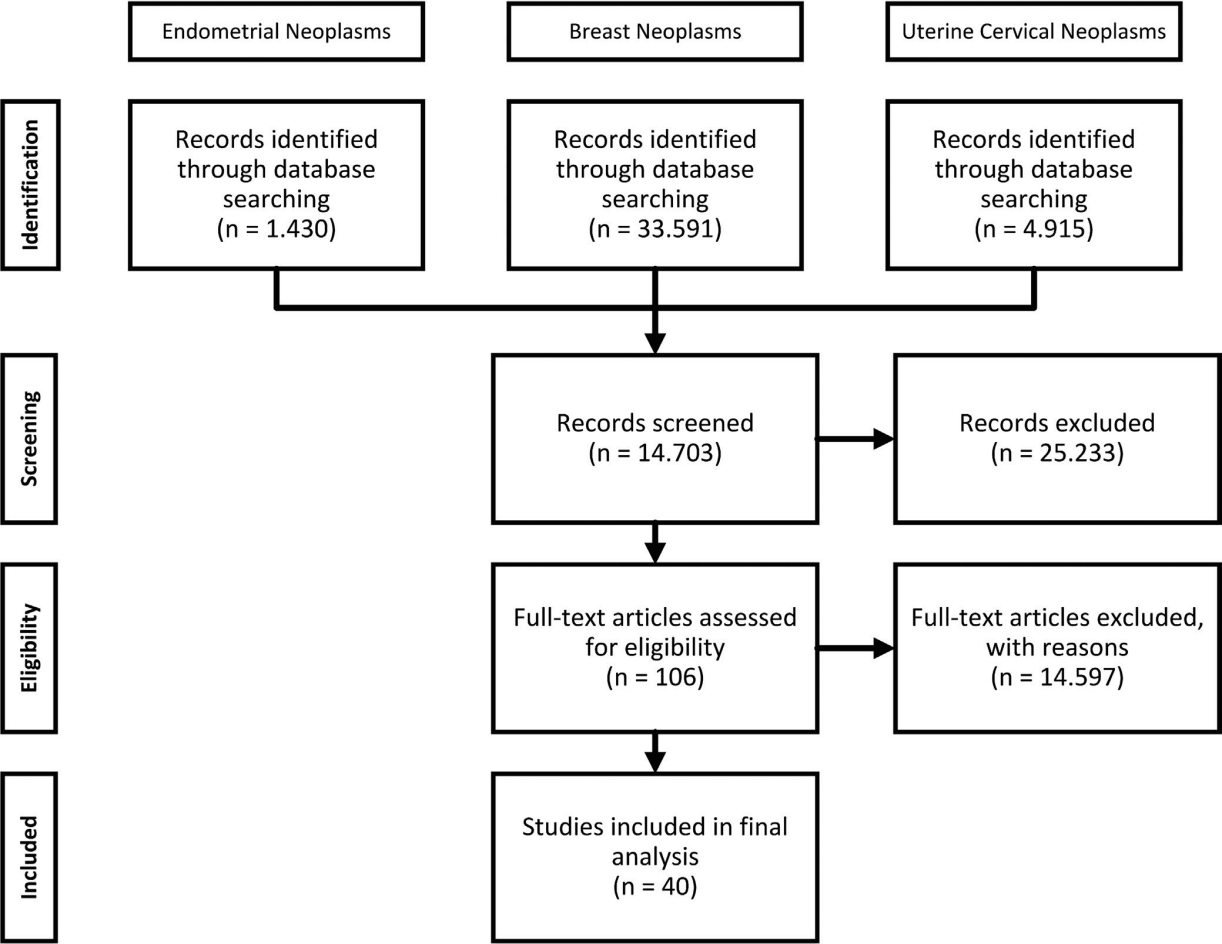
PRISMA diagram of the conducted search inquiries

A literature review was conducted via the biggest medical literature databases (MEDLINE, PubMed, ScienceDirect) to obtain studies related to gynaecological CCLs. The employed search terms in the form of keywords were “breast cancer cell lines”, “endometrial cell lines”, “cervical cancer cell lines”, “Gynaecological cancer cell lines”. Used MeSH identifiers were “Breast Neoplasms”, “cell line”, “cell lines, tumor”, “cell line, transformed”, “uterine cervical neoplasms” and “endometrial neoplasms”. With the help of this search algorithm and specific filters (5 years, human, review), we were able to find relevant new impactful studies on gynaecological CLs (Table 1). We specifically searched for the corresponding originators’ study which was crosschecked via the Cellosaurus database. The search inquiries and presentation of the final number of included studies after exclusion and filtering is presented in Figure 2 and has been prepared in accordance with the PRISMA guidelines for review articles.

**TABLE 1:** Overview of the preformed search results

Search terms	Results
("Breast Neoplasms"[Mesh]) AND "Cell Line, Tumor"[Mesh] with filters (5‐year filter; review, human)	No. 96
("Cell Line, Tumor"[Mesh]) AND "Endometrial Neoplasms"[Mesh] (5‐year filter; review, human)	No. 3
("Cell Line"[Mesh]) AND "Uterine Cervical Neoplasms"[Mesh] (5‐year filter; review, human)	No. 7
("Uterine Cervical Neoplasms"[Mesh]) AND "Breast Neoplasms"[Mesh]) AND "Endometrial Neoplasms"[Mesh]) AND "Cell Line"[Mesh] (5‐year filter; review, human)	No. 1

## 3 BREAST CANCER AND ITS CELL LINES

### 3.1 Introduction

The second most common cancer worldwide is breast cancer. This type of cancer is ranked first in incidence in women living either in developed or developing countries (Table 2). It is ranked as the fifth common cause of cancer death (627 000 deaths/per year).^14^ A short overview of the most important histological subtypes is shown in Table 3.

**TABLE 2:** Epidemiological worldwide cancer statistics for 2018

Estimated number of incident cases and deaths worldwide, both sexes, all ages (2018)			
No.	Cancer	Incidence	Mortality
1	Lung	2.093.876	1.761.007
2	Breast	2.088.849	626.679
3	Colorectum	1.849.518	880.792
4	Prostate	1.276.106	358.989
5	Stomach	1.033.701	782.685
6	Liver	841.080	781.631
7	Oesophagus	572.034	508.585
8	Cervix uteri	569.847	311.365
9	Thyroid	567.233	41.071
10	Bladder	549.393	199.922

Estimated number of incident cases and deaths worldwide, females, all ages (2018)			
No.	Cancer	Incidence	Mortality
1	Breast	2.088.849	626.679
2	Colorectum	823.303	396.568
3	Lung	725.352	576.060
4	Cervix uteri	569.847	311.365
5	Thyroid	436.344	25.514
6	Corpus uteri	382.069	89.929
7	Stomach	349.947	269.130
8	Ovary	295.414	184.799
9	Liver	244.506	233.256
10	Non‐Hodgkin lymphoma	224.877	102.755

Estimated age‐standardized incidence and mortality rates (World) in 2018 (Low income, Low‐middle income, females, all ages)			
No.	Cancer	Incidence	Mortality
1	Breast	31.1	14.9
2	Cervix uteri	17.5	11.5
3	Colorectum	6.3	4.1
4	Ovary	5.8	4
5	Lung	4.5	4.1
6	Corpus uteri	3.8	1.3
7	Stomach	3.7	3.3
8	Liver	3.4	3.3
9	Lip, oral cavity	3.2	2.4
10	Thyroid	3.2	0.54

Estimated number deaths worldwide, both sexes, all ages (2018)		
No.	Cancer	Mortality
1	Lung	1.761.007
2	Colorectum	880.792
3	Stomach	782.685
4	Liver	781.631
5	Breast	626.679
6	Oesophagus	508.585
7	Pancreas	432.242
8	Prostate	358.989
9	Cervix uteri	311.365
10	Leukaemia	309.006

Data summarized from the Globocan database.^14^

**TABLE 3:** Breast cancer subtypes

Non‐invasive histological types	
Adenocarcinoma (99.9%)	Ductal carcinoma in situ (DCIS) (80.1%)
	Lobular carcinoma in situ (LCIS) (15.9%)
	Intraductal and lobular in situ carcinoma (3.4%)
	Other adenocarcinomas (0.5%)
Other in situ histologies (0.1%)	

Invasive histological types^a^		
Carcinoma (99.5%)	Adenocarcinoma (97.7%)	Infiltrating duct carcinoma (72.4%)
		Lobular carcinoma, NOS (9.9%)
		Mixed NST and special subtypes (9.7%)
		Mucinous adenocarcinoma (1.8%)
		Other adenocarcinomas (1.5%)
		Papillary adenocarcinoma (0.8%)
		Adenocarcinoma NOS (0.6%)
		Tubular adenocarcinoma (0.5%)
		Paget disease (0.3%)
		Inflammatory adenocarcinoma (0.2%)
	Unspecified, carcinoma, NOS (0.9%)	
	Other specific carcinoma (0.8%)	
	Epidermoid carcinoma (cca. 0.2%)	
Sarcoma and soft tissue tumours (0.1%)	Hemangiosarcomas (cca. 0.1%)	
	Other sarcomas (cca. 0.1%)	
Other specific types (0.2%)	Phyllodes tumour, malignant (0.2%)	
	Other (0.0%)	
Unspecified (0.2%)		

Intrinsic subtype	IHC status	Grade	Prevalence
Luminal type A	(ER+, PR+), HER2‐, Ki‐67‐(<15%)	1/2	23.7%
Luminal type B	(ER+, PR+), HER2‐, Ki‐67+(>15%) (ER+, PR‐), HER2‐, Ki‐67+(>15%)	2/3	38.8% 14%
HER2	(ER‐, PR‐), HER2+	2/3	11.2%
Basal	(ER‐, PR‐), HER2‐, basal markers+	3	12.3%
Normal like	(ER+, PR+), HER2‐, Ki‐67‐ (<15%)	1/2/3	7.8%

Abbreviations: IHC, immunohistochemical status; ER, oestrogen receptor; PR, progesterone receptor. Cut‐off value for Ki‐67 is 15%. Summarized from.^31^, ^58^, ^78^

^a^ Summarized from the SEER database.

#### 3.1.1 Invasive breast cancer

We distinguish multiple different types of invasive breast cancer (IBC). The distinctions are based on the molecular as well as histopathological properties.^15^ In a routine diagnostic procedure, multiple different criteria will be gathered and analysed; tumour size and grade (Elston and Ellis); presence of lymphovascular invasion; DCIS and LCIS; assessment of surgical margins; the immunohistochemical profile of hormonal receptors (eg oestrogen – ER (most commonly the alpha receptor), progesterone – PR), the HER2 receptor status and analysis of proliferation index, determined with the Ki‐67 (Mib 1) percentage of nuclear expression, is an additional independent prognostic parameter for DFS and OS in BC patients.^16^ Furthermore, proteases and genetic profiling can be of great help in determining the need for additional treatment. These features are not only of great importance for the clinician but also of great importance for the researcher during CL culturing. Namely, for successful culturing, the scientist must know the specific properties to provide and use an adequate environment, culturing methods, characterization methods, etc, to preserve the characteristics of the source tissue. Primary BC can be classified into two categories. The first are the non‐invasive BCs. These are ductal carcinoma in situ (DCIS) and lobular carcinoma in situ (LCIS). These types of cancer are confined within the milk ducts and lobules of the breast and surrounded by an intact basement membrane and myoepithelial cells (Table 3).^16^ More common are invasive BC types. The term invasive breast carcinoma (IBC) refers to a large heterogeneous group of malignant epithelial neoplasms of the breast.

Due to treatment purposes, distinct outcomes and responses to therapy, all IBCs are grouped into the following subtypes: (a) ER‐positive, HER2‐negative; (b) ER‐positive, HER2‐positive; (c) ER‐negative, HER2‐positive; (d) ER‐negative, HER2‐negative.^16^ The majority of BCs are unifocal and can occur in any quadrant of the breast, with a higher frequency in the upper outer quadrant. A synchronous contralateral tumour is found in approximately 2% of patients. The macroscopically most common features in advanced stages of IBC are skin retraction, nipple inversion, nipple discharge, change of texture or colour of the skin. Otherwise, lesions that are discovered early are mostly asymptomatic and may clinically show themselves on palpation as a lump. About 5.15% of all palpable BCs are not seen on a mammogram but can be identified with ultrasound. MRI is the most sensitive method.^16^

The most common is the invasive carcinoma of no special type (NST), which accounts for 40%‐75% of all invasive BC types and is commonly present alongside DCIS. The special morphological patterns include a variety of different patterns. These BCs can be mixed IBC‐NST and special subtypes such as the pleomorphic carcinoma, BC with osteoclast‐like giant cells, carcinoma with choriocarcinomatous features and IBC with melanocytic differentiation, oncocytic pattern, lipid‐rich pattern, glycogen‐rich clear cell pattern and sebaceous pattern.^16^ To continue, based on epidemiological data, invasive lobular carcinoma (ILC) comes in second place.^16^ It presents itself in the somewhat older female population (57‐65 years) and accounts for 5%‐15% of all invasive BCs. ILC is rather heterogeneous due to its various subtypes that are based on the histological picture. The most common picture (‘classic form’) is comprised of small uniformly looking cancer cells. These cells grow in single file, linear pattern and invade the stroma.^16^ Two very rare types of BC are the tubular carcinoma and cribriform carcinoma (1.6% and 0.4%). Despite their rarity, they have a very good prognosis.^16^

Mucinous carcinoma presents approximately 2% of all BCs cases. This type is more common in women older than 55 years (median 71 years) and has a good 5‐year disease‐free survival rate as well as a very low local recurrence rate. The metaplastic carcinoma, also known as carcinosarcoma (if the mesenchymal component is malignant), is known for its heterogeneity and different components that arise from neoplastic differentiation. Its characterization can be done based on its morphology that is marked by the presence of squamous cells and/or mesenchymal‐looking elements (cartilage, spindle cells, bone, etc). These present 0.2%‐1% of all BC cases and less frequently metastasize into axillar lymph nodes in comparison with invasive NST carcinomas. Other even rarer types of invasive BCs are micropapillary carcinoma, salivary gland/skin adnexal type tumours, adenoid cystic carcinoma, carcinoma with apocrine differentiation, invasive carcinomas with neuroendocrine differentiation, papillary carcinoma and inflammatory carcinoma.^16^

In 2019, the new WHO classification for breast cancer was published.^16^, ^17^ It includes a number of changes. To briefly summarize the most important changes: (a) carcinoma with medullary features, which was previously a separate entity is now regarded as a tissue infiltrating lymphocyte (TIL)‐rich IBC‐NST; (b) oncocytic, lipid‐rich, glycogen‐rich clear cell, sebaceous, pleomorphic, melanotic, oncocytic and choriocarcinomatous carcinomas, carcinoma with osteoclast‐like giant stromal giant cells, previously separate entities, are now regarded as rare variants of carcinoma NST; (c) inflammatory, bilateral and non‐synchronous breast carcinomas, previously separate entities, are now recognized as distinct clinical presentations rather than special subtypes; (d) lobular carcinoma in situ consists now of classic, pleomorphic and florid types; (e) addition of neuroendocrine neoplasms group, of which true neuroendocrine neoplasms are typed as neuroendocrine tumour (NET), small‐cell neuroendocrine carcinoma, large‐cell neuroendocrine carcinoma; (f) neuroendocrine differentiation was overridden by morphological tumour type (NST, mucinous, solid papillary); (g) well‐differentiated liposarcoma in phyllodes tumours is no longer a histological criterion of malignancy by itself; (h) mucinous cystadenocarcinoma is a new recognized entity; (i) a previously known breast tumour with resemblance to the tall variant of papillary thyroid carcinoma but now categorized and grouped under the name tall cell carcinoma with reversed polarity; (j) periductal stromal tumour is no longer a separate entity but a variant of the phyllodes tumour; (k) mesenchymal tumours, haematolymphoid tumours and genetic tumour syndromes are now covered in dedicated chapters.^16^, ^17^

### 3.2 Breast cancer cell lines

The field of BC CLs has been established with the first BC CL from Lasfargues and Ozzello in 1958 called BT‐20.^18^ Following an increased interest in this area, researchers have, in the following years, isolated and presented an increasing number of CCLs. We face a growing assortment of different CCLs, whereas the integrity and uniqueness of certain CCLs are doubtful. The most commonly used BC CL is still MCF7, T47D and MDAMB231.^4^, ^19^ Their properties can be seen in Table 4. We also published some more information on the less known BC CLs in one of our previous studies.^4^

**TABLE 4:** Properties and names of the most commonly used BC CL

Name	Properties	Type
MCF7	ER+/PR+/HER2‐	Luminal type A
MDAMB231	ER‐/PR‐/HER2‐	Triple negative
SkBr3	ER+/PR+/HER2+	HER2 positive
T‐47D	ER+/PR+/HER2‐	Luminal type A
BT‐20	ER‐/PR‐/HER2‐	Triple negative

Molecular subtypes of cell lines		
HER2	ER‐negative and HER2‐positive profile Over‐represented genomic profile on the chromosomal region 17q12 Present based on their profile, a bridge between luminal and basal cell lines Heterogeneous with luminal and basal features.	
LUMINAL	Expression of ER and or PR miRNA specific profile (eg hsa‐miR‐501‐5p, hsa‐miR‐202, hsa‐miR‐760 and hsa‐miR‐62) A subdivision into A (luminal A‐like; ER+, PR+, HER2‐, Ki‐67 low) and B (liminal B‐like; ER+, HER2‐; Ki‐67 high or PR low/‐; luminal B‐like, HER2 + m ER+, Ki‐67 any, PR any) Good differentiation, more intercellular tight junctions.	
TNBC	TNBC basal A	Common cytokeratins (KRT4/5/6A/6B/13/14/15/16/17) Integrins (ITGA6, ITGB4/6) Specific miRNA Better differentiation
	TNBC basal B	Higher expression of genes that promote aggressive behaviour (eg vimentin, moesin, plasminogen‐activating factor) CD44 + and CD24‐ Specific miRNA Worse differentiation

Note

Summarized based on the Cellosaurus database.^12^

The first to successfully culture a BC CL was Lasfargues and Ozzello in 1958. The CL was named BT‐20.^18^ The tissue was obtained from a 74‐year‐old patient, who had a mammary duct‐cell carcinoma of no special type. The cell collection was performed via sampling of the spillage that occurred during tumour preparation (slicing).^18^ Certain characteristics of the CL are as follows: triple‐negative breast cancer (TNBC) with a basal A subtype ^20^; homozygous CDKN2A deletion, homozygous point mutation of TP53 p. Lys132Gln (c.394A>C) and RB1 p. Ile388Ser (c.1163T>G); heterozygous point mutation of RB1 p. Pro515Leu (c.1544C>T) and PIK3CA p. His1047Arg (c.3140A>G) as well as PIK3CA p. Pro539Arg (c.1616C>G).^21^

MCF7 is one of the most studied CLs in the world. It was derived from the free‐floating cells of the primary CL 734B. The latter was obtained from a patient with metastatic BC. Specifically, the cells were sampled from a malignant pleural effusion. The patient was a 69‐year‐old Caucasian female (blood type 0, Rh‐positive) named Sister Catherine Frances (Helen Marion).^22^, ^23^ It has been reported that she underwent surgery for both breasts. At first, a mastectomy was performed to remove a benign tumour in her right breast. Later a radical mastectomy of her left breast was performed due to an adenocarcinoma. This happened 7 and 3 years, respectively, before initiating the primary culture. The CL was first described by Soule HD et al in 1973 and had been, as described by the authors, then maintained for 3 years.^22^

The authors described that the primary CL exhibited a typical rapid proliferation that is common for most metastatic tumours in vitro. The primary cells (large, immature, striated) did microscopically not appear to be epithelial in origin. It was concluded that they may have been mesothelial in origin or abnormal fibroblasts, due to morphological characteristics and collagenization of the cultures.^22^ Certain characteristics of the CL: ER positivity (first described by Lippman and Horwitz)^24^, ^25^; PR heterogeneity, which is generally attributed to the coexistence of several sublines, each possessing different stages of differentiation as well as dependent on the cell cycle phase and population doubling time (PDT)^26^; in microarray profiling, the cl genome clusters with the BC luminal type A type; HER2 negativity; additional expression of androgen and glucocorticoid receptors.^23^, ^27^ However, there have been reports on successful subculturing and generations of MCF‐7 cells overexpressing HER2. This CL and the information it presents was referenced in many studies.^23^, ^28^

MDAMB231 is another BC CL that was isolated from a pleural effusion in a BC patient. The CL had been together with MDAMB134 (mean chromosome number 43) and MDAMB175 (mean chromosome number 49) first described in 1974 by Cailleau et al.^29^ The CL was cultured from a single sample of pleural effusion obtained on 17 October 1973. The patient was a 51‐year‐old Caucasian woman who had had a right radical mastectomy in January 1969 for a poorly differentiated invasive ductal carcinoma. The authors reported that the patient developed a pericardial (June 1973) and left sided pleural effusion (July 1973). The effusions were of metastatic origin due to a BC *primum*. Subsequently, oophorectomy and systemic treatment were initiated. At first, she was given 5‐FU and prednisone. Later, in September 1973, combined chemotherapy (cyclophosphamide, adriamycin and amethopterin) was started. However, the patient's state deteriorated, and she died on 13 January 1974.^29^ The pleural effusion from which the CL was cultured occurred after systemic treatment. Certain characteristics of the CL are as follows: epithelial growth pattern; a near triploid chromosome number (from 60 to 70); ER, PR and E‐cadherin–negative status; heterozygous point mutation for BRAF p. Gly464Val (c.1391G>T) and KRAS p. Gly13Asp (c.38G>A), homozygous point mutation for TP53 p. Arg280Lys (c.839G>A) and CDKN2A gene deletion; basal B subtype.^30^, ^31^

In the last years, many studies have analysed the field of BC CLs. A large‐scale study was done by Xiaofeng et al, who not only offered a detailed overview of 92 BC CLs and their molecular classification (luminal A, luminal B, HER2‐positive and triple‐negative subtypes divided into basal A and basal B) but also highlighted inconsistencies in studies with regard to primary marker status reports.^31^ The authors came to several conclusions. Firstly, as an observation, the genetic and epigenetic categorization between BC CLs and primary tissue is not one to one. Secondly, TNBC CLs can be genetically subdivided into basal A and B groups that have specific properties (eg phenotype, molecular properties). Namely, the TNBC A subtype is supposedly characterized by the expression of basal keratins (KRT4/5/6/13/14/15/16/17). This expression profile shows similarity with the core basal tumours. The TNBC B subtype has a characteristic stem cell profile of CD44+CD24‐ and migration markers such as vimentin. This shows promise for modelling claudin—low and/or metaplastic breast cancers.^31^

The study from Holiday et al 2011 was, at that time, one of the rare and most comprehensive studies on this specific subject at that time. Nine years after their study, we can see that although there is now a reasonable number of BC CLs, these still lack some of the rarer histopathological types (eg phyllodes tumours, male BC CLs and inflammatory BC). Another recent study, done by Lima Mota et al, focused on the molecular characterization of BC CLs by clinical immunohistochemical markers.^32^ The authors set out to evaluate the hormone receptor and HER2 receptor expression and classify the BC CL based on the molecular subtypes. The used BC CLs were as follows: SKBR3, MCF‐7, MCF‐7/AZ, Hs578T, MDA‐MB‐231, MDA‐MB‐468, BT‐20 and T47D. What is surprising is that their results differ from previously reported CL expression profiles for BT20 CL. This CL has been in a wide variety of studies described as a TNBC BC CL.^12^, ^30^, ^31^, ^33^ However, the authors report results of it being HER2 overexpressed.^32^ Other BC CLs that have inconsistent expression profiles in various studies are HCC1007, HCC1419, HCC1500, HCC2185, SUM52PE, SUM44PE, EVSA‐T and EVSA‐T.^20^, ^31^, ^34^, ^35^, ^36^, ^37^ The reasons for these inconsistencies can be several. We will give an overview of this topic in the last segment of the paper.

## 4 CANCER OF THE ENDOMETRIUM AND ITS CELL LINES

### 4.1 Endometrial cancer

EC presents a very common gynaecological malignancy. It has a yearly incidence rate of 60 000 (United States) new cases and more than 10 000 deaths.^38^, ^39^, ^40^, ^41^ On a global scale, there are 382 069 new cases yearly and 89 929 deaths.^38^, ^39^, ^40^, ^41^ The incidence of this cancer is very tightly linked to certain epidemiologic factors. According to literature, obesity being one of the most important.^38^, ^39^, ^40^, ^42^ Others include the exposure to unopposed oestrogens or tamoxifen, diabetes, nulliparity, early‐onset menarche and late‐onset menopause. In one of our previous publications, we provided an in‐depth overview of EC CLs.^2^

We can, based on the latest WHO classification (2020, 5th), divide EC into multiple histological subtypes. The most common is the endometrioid type (up to 80%) followed by mixed cell type (up to 10%), serous (up to 10%); carcinosarcoma (<10%), clear cell (<10%), undifferentiated and dedifferentiated carcinomas (<10%), mixed carcinoma (<10%) and other types (<10%; mesonephric adenocarcinoma, squamous cell carcinoma NOS, mucinous carcinoma, intestinal type and mesonephric‐like adenocarcinoma).^43^, ^44^, ^45^ The endometrioid carcinomas are graded with the use of the FIGO classification system. The grades are determined by the percentage of solid growth patterns (Grade 1: <5%; Grade 2: 6%‐50%; Grade 3: >50% solid growth). Endometrioid types have commonly a positive hormone receptor status (ER, PR) and p53 wild‐type (G1/2) or heterogenous status (G3). Microscopically one can observe tall columnar cells lining back‐to‐back glands without intervening stroma. The growth pattern can also be cribriform. The microscopist has to be careful to not include areas of squamous differentiation when assessing the percentage of solid growth and consequently the grade. Serous carcinomas have a positive hormone receptor expression (ER, PR), are of the abnormal p53 (p53abn) molecular subtype and have a p16‐ and PTEN‐positive status. Histological features include tumour cells (nuclear atypia, mitotic figures) that form papillary structures. The glands have serrated outlines. Serous carcinomas are frequently clinically occult, often invade the lymphovascular space and have therefore a worse prognosis. Clear cell carcinomas are uncommon. They can have an either negative or positive receptor status (ER, PR) p53 heterogenous status, variable PTEN and p16 status (±) and show positive staining for Napsin A, Racemase and HNF1ß. These tumours share many morphologic features with ovarian clear cell carcinomas. The different architectural features include a combination or solely a solid, glandular or papillary architecture. Also, commonly cells with abundant clear cytoplasm can be observed.^45^, ^46^

Based on The Cancer Genome Atlas (TCGA), endometrial carcinomas are classified into four subgroups. These are as follows: (a) the POLE (DNA polymerase ε) ultramutated group, (b) the hypermutated/microsatellite unstable (MSI) group, (c) the copy number low/microsatellite stable group and d) the copy number high (serous‐like) group.^44^, ^47^ The routine of identifying and correctly specifying the molecular EC groups can be done by following a system known under the name of Proactive Molecular Risk Classifier for Endometrial Cancer (ProMisE). The first step is identifying specimens with POLE pathogenetic mutations (‘group a’). If the specimen has a non‐pathogenetic mutation or a wild type, then mutation mismatch repair proteins (MMR) are inspected (unstable – ‘group b’). In specimens with intact MMR, immunohistochemical p53 staining is performed. This shows either an aberrant (corresponding with ‘group d’) or wild‐type variant (‘group c’).^48^

For a long time, EC was classified into two categories which correlated to its aggressiveness. With the advancement of technology and new studies, the molecular background became much more important due to its implications in prognosis. Therefore, the newest WHO classification (2020, 5th) includes explanations on the new molecular classification system for ECs and how it relates to the traditional histomorphologic evaluation. The characteristics of group 1 are as follows: more common, lower risk, dependent on oestrogen, hormone receptor (mostly) positive (ER, PR), good prognosis, comparatively younger population (between 55 and 65), endometrioid histology (grade 1/2) and with most commonly no specific molecular profile (sometimes mismatch repair deficient—MMRd). The characteristics of group 2 are as follows: no growth dependency on oestrogen, mostly hormone receptor negative, older population of females (over 65), bad prognosis, histologic clear cell or serous carcinoma with respectively no specific molecular profile or abnormal p53 status. Grade 3 endometrioid EC is best considered as a separate category since it can have any of the molecular anomalies and be therefore in any group.

The standard treatment for endometrial cancer consists of primary hysterectomy and bilateral salpingo‐oophorectomy (eg laparoscopic or robotic). The 5‐year overall survival ranges from 74% to 91% in patients without metastatic disease.^49^

### 4.2 Endometrial cancer cell lines

Endometrial CCL that is widely used is Ishikawa, HEC‐1‐A, HEC‐1‐B and KLE. Some other examples of commonly described immortal endometrial CLs include HES and hTERT‐EEC.^2^ What is more, reports said that the CLs are commonly contaminated with the MCF‐7 cancer cells and the HeLa CL.^50^ Other issues include the misidentification and redundancy of these cell isolates. This was shown by Korch et al in 2012,^50^ who presented proof that ECC‐1 isolates were contaminated and genotyped either as Ishikawa cells, MCF‐7 breast cancer cells (or a combination).^2^ Furthermore, another issue is the uncertainty regarding their primary type (I or II) due to conflicting reports in the literature.^8^, ^51^, ^52^ Among the studies that tackle the issue of EC CLs some of them show similarities while others differ in certain aspects.^8^, ^9^, ^50^, ^51^, ^53^ Our findings from the literature were that HEC‐1‐A, HEC‐1‐B and KLE are reported differently.^2^ Crucial properties and the originators' information of the most common EC CLs are succinctly summarized in Table 5.

**TABLE 5:** Properties of the most common EC CL

Cell line name	HEC‐1‐A and HEC‐1‐B	Ishikawa	AN3‐CA	KLE
First described	Kuramoto H in 1972	Nishida et al in 1985	Dawe CJ et al in 1964	Richardson et al in 1984
Patient	71‐year‐old woman	39‐year‐old woman	55‐year‐old woman	64 to 68‐year‐old female
Tumour	Endometrial adenocarcinoma	Endometrial adenocarcinoma	Uterine neoplasm associated with the clinical syndrome of malignant acanthosis nigricans, obtained from lymph node	Tissue of a colon metastasis from a poorly differentiated G3 endometrial adenocarcinoma
Chromosomes	HEC‐1A – diploid. HEC‐1B – tetraploid	Diploid chromosomal range	Diploid chromosomal range	‐
Special remarks	HER‐1‐A (parent) HEC‐1‐B (child) Heterozygous point mutation KRAS p. Gly12Asp (c.35G > A); homozygous point mutation for TP53 p. Arg248Gln (c.743G > A) and HEC‐1‐B no PTEN mutation	ER and PR disappear after long‐term culture	MSI instability; Heterozygous point mutation of MAPK3 p. Pro373Ser (c.1117C > T), heterozygous point mutation of PIK3R1^a^ and heterozygous point mutation of TP53 p. Gly389Trp (c.1165G > T); homozygous point mutation of PTEN p. Arg130Glnfs*4 (c.389delG); KRAS wild type	PTEN, KRAS wild type, no mutation Low MSI
Literature	79, 80, 81	82, 83	^84^	9, 12, 85

Cell line name	HEC‐1‐A	HEC‐1‐B	Ishikawa	AN3‐CA	KLE
Histopathological properties	Status	Status	Status	Status	Status
ER	Low	Low	High	Low	low
PR	Low	Low	High	Low	Low
PTEN	High	High	Low	Low/Deletion	High
Grade	G2	G2	G1	Metastasis	Metastasis
Type	II	II	I	I	II

Note

Summarized from the Cellosaurus databank (Bairoch et al^12^).

^a^ Due to space issues: p. Arg557_Lys561delArgGluIleAspLysinsGln (c.1670_1681delGAGAAATTGACA).

## 5 CERVICAL CANCER AND ITS CELL LINES

### 5.1 Cervical cancer

The last cancer type to be discussed in this review is uterine cervix cancer (CC). CC is a common type of cancer and has a relatively high mortality among gynaecological cancers with stark regional differences. The incidence and death toll are on a global scale estimated at approximately 570 000 and 311 000 per year.^54^ Interregional differences in mortality can be attributed to countries lacking cervical cancer screening and prevention programmes, since this type of cancer remains the second most common type (17.8 per 100 000 women) as well as the cause of cancer deaths (9.8 per 100 000) among all types of cancer in women that live in lower income countries (Table 2).^14^ Furthermore, collectively, most of CC cases (80%‐90%) occur in these countries (eg parts of Africa and Asia).^55^ The most important directly linked factor for CC is the human papillomavirus (HPV) infection,^56^ which is also the most common sexually transmitted disease. Through research, more than 200 different types have been found, identified and systematically classified into 5 genera (α, β, γ, μ and ν).^57^ Based on their oncogenic potential, these are labelled as high risk and low risk. The most studied types are of the α genera, since these have been directly linked to almost all squamous intraepithelial lesions and cancers of the cervix and anus as well as to a subset of penile, vulvar and vaginal cancers.^57^ HPV types and their biologic potential can be seen in Table 6. It has been estimated that HPV is responsible for almost a tenth of human malignancies (7%‐8%). It is associated with almost all cases of CCs (96%) and anal cancers (93%). Furthermore, almost two thirds of all vaginal cancers (64%) and oropharyngeal carcinomas (63%) arise due to its oncogenic potential. And lastly, HPV also presents an important factor in the development vulvar cancer (51%) and penile cancers (36%).^57^

**TABLE 6:** HPV characteristics

HPV types and their biologic potential	
Low‐risk (non‐oncogenic) types	HPV 6, 11, 40, 42, 43, 44, 54, 61, 72, 81
High‐risk (oncogenic or cancer‐associated) types	HPV 16, 18, 31, 33, 35, 39, 45, 51, 52, 56, 58, 59, 68, 69, 82
Squamous cell carcinoma	HPV 16 (59%), 18 (13%), 58 (5%), 33 (5%), 45 (4%)
Adenocarcinoma	HPV 16 (36%), 18 (37%), 45 (5%), 31 (2%), 33 (2%)

HPV‐associated diseases	
Disease	HPV type association
Cutaneous warts	1, 2, 3, 4, 27, 57
Anogenital warts	6, 11, 53
Mucosal cancers	16, 18, 31, 33, 35, 39, 45, 51, 52, 56, 58, 59, 66, 68, 73, 82
Non‐melanoma skin cancers	1, 5, 8, 9, 17, 20, 23, 38
Bowen disease	16, 18, 31, 32, 34
Epidermodysplasia verruciformis	5, 8, 9, 12, 14, 15, 17, 19‐25, 36‐38, 46, 47, 49, 50

Clinical properties	
Signs	Irregular or heavy vaginal bleeding; post‐coital bleeding
Risk factors	Early onset of sexual activity, multiple sexual partners, a high‐risk sexual partner, history of sexually transmitted infections, history of vulvar or vaginal neoplasia, immunosuppression.

Note

Summarized after.^61^, ^62^, ^63^

The most common histologic types of CC are squamous cell carcinoma (64.5%), adenocarcinoma (28.9%) and 6.6% other histology's.^58^, ^59^ Based on a recent study from 2019, HPV infection may also be implicated in developing some types of breast cancer.^60^ The presence of screening programmes for cervical cancer and public health programmes for HPV vaccination and education of the general public led, according to sources, to a significant decrease in the incidence and mortality of cervical cancer over the past 50 years in developed countries (75%).^61^, ^62^, ^63^

It is worth noting that viral load seems to be in an inverse correlation with the malignancy of the lesion. This can be observed predominantly in non‐melanoma skin cancer (NMSC; cutaneous lymphomas, adnexal tumours, Kaposi's sarcomas, Merkel‐cell carcinomas, basal cell carcinomas—BCCs, squamous cell carcinomas—SCCs) and other skin lesions, which is in contrast to the direct carcinogenic effect of genital HPVs.^57^, ^64^ In such cases, this evidence supports a hit‐and‐run mechanism of carcinogenesis.^64^ Treatment depends on the extent of the disease. The more advanced cases may require radical hysterectomy or chemoradiation, or even a combination of both. At the same time, conservative, fertility‐preserving surgical procedures have become the standard of care for women with low‐risk, early‐stage disease.^55^ Based on the information from copy number variation (CNV), methylation, mRNA and miRNA profiles, cervical cancer has three distinct molecular subtypes of CC: SCC keratin—high, SCC keratin—low and adenocarcinoma.^65^ The differences between these include the following: enriched expression of some genes (eg PIK3CA, ADH7 and SPRR3) in the SCC keratin—high compared with the SCC keratin—low cluster, more frequent CNVs, including common EGFR amplification in SCCs, a high number of aberrations in tumour‐suppressor genes related to TGF‐β pathway in adenocarcinomas including SMAD4 and TGFBR2 deletions, and increased DNA methylation in adenocarcinomas.^65^

### 5.2 Cervical cancer cell lines

**FIGURE 3:**
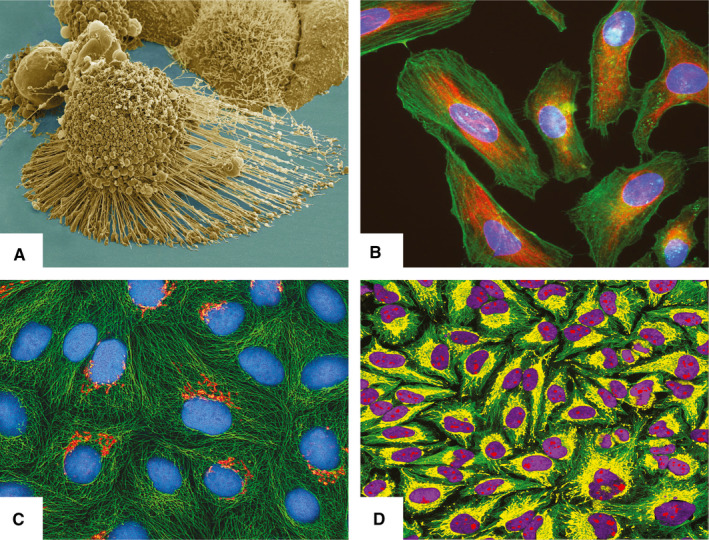
HeLa cell line. (A) Electron micrograph of an apoptotic HeLa cell (source: National Institutes of Health—NIH; https://imagebank.nih.gov/details.cfm?imageid=1463); (B) Immunofluorescence image of HeLa cells grown in tissue culture and stained with antibody to actin in green, vimentin in red and DNA in blue (source: GerryShaw—wikimedia.commons; https://commons.wikimedia.org/wiki/File:HeLa_cells_stained_with_antibody_to_actin_(green)_,_vimentin_(red)_and_DNA_(blue).jpg); (C) Multiphoton fluorescence image of cultured HeLa cells with a fluorescent protein targeted to the Golgi apparatus (orange), microtubules (green) and counterstained for DNA (cyan) (source: NIH; https://commons.wikimedia.org/wiki/File:HeLa‐I.jpg); D) Immunofluorescence of HeLa cells showing microtubules in green, mitochondria in yellow, nucleoli in red and nuclear DNA in purple (source: GerryShaw—wikimedia.commons; https://commons.wikimedia.org/wiki/File:HeLa‐Tubulin‐HSP60‐Fibrillarin‐DNA.jpg). All material is published under the CC license or is in the public domain

**FIGURE 4:**
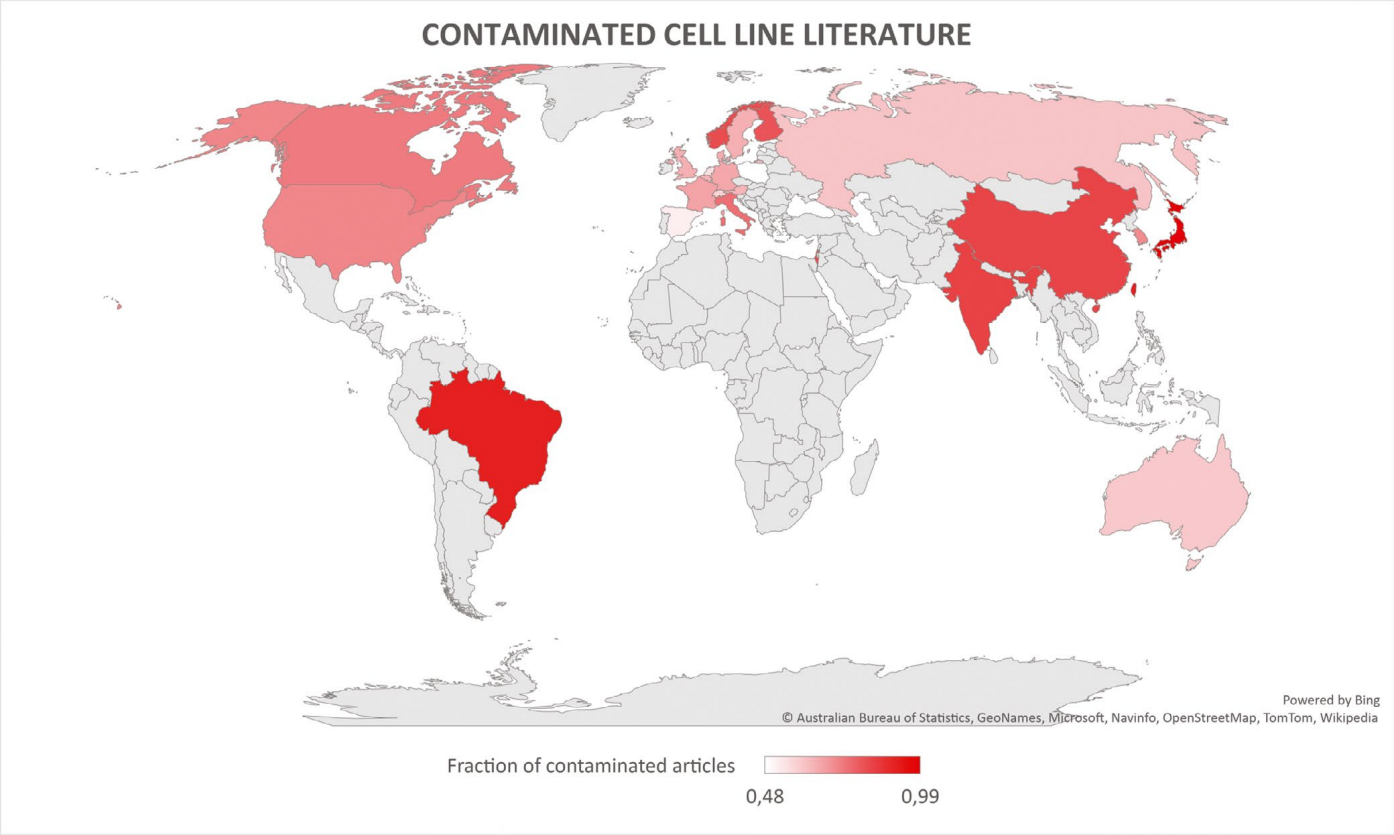
A simplified geographical overview of the percentage of contaminated primary articles as a fraction of the total number of articles on cells per country (Top 5: Japan, Brazil, Taiwan, India, China). Adapted from the dataset from Horbach and Halffman^68^ under the CC license

The most famous and first CCL in the world is HeLa (Figure 3). The HeLa cells are named after the 30‐year‐old patient Henrietta Lacks who died in 1951 due to an aggressive adenocarcinoma of the cervix.^66^ Some of the tissue obtained from a cervical biopsy was supplied to the Tissue Culture Laboratory in the Department of Surgery at The Johns Hopkins Hospital for research purposes. Contrary to their previous results, the cells grew robustly and became the first human CCL immortalized in tissue culture. As is widely known, the naming process consisted of utilizing the initial 2 letters of Henrietta Lacks' first and last names.^66^ The cells have been shown to contain human papillomavirus (HPV) 18 DNA, and HPV18‐positive HeLa cells have been linked to changes in microRNA expression. These results were obtained by Gey and colleagues, which published their study in 1952.^67^ The most common cervical CCLs are shown in Table 7. What is important to note is the fact that these cells have been in circulation for more than 60 years. Subsequently, they are almost ubiquitous.^68^ This has, through the years, also led to many contaminations and has become a crucial problem in CL culturing. This furthermore led to an accumulation of articles and studies that reported their research on contaminated or mutated CLs. The authors Horbach and Halffman were able to identify 32 755 such articles (up to the year 2017).^68^ For their search, the authors utilized two methods. One was via the cross‐search of the CLs documented in the eight versions of the International Cell Line Authentication Committees (ICLAC) list of misidentified CLs and by using the WoS database for all articles stating the names of one of the 451 listed CLs.^68^ Figure 4 shows, based on the authors published data from 2017, a simplified geographical overview of the areas with the highest percentage of contaminated primary articles as a fraction of the total number of articles on cells per country (primary data set is available at the primary authors publication).^68^ The authors utilized the ICLAC list of misidentified cells. The latest version is version 10 and was released 25 March 2020. The register currently lists 552 cell lines.^69^ Besides the importance of HPV status, karyotyping, p53 and pRB expression, a recent study compared the secretomes of different cervical CCLs and highlighted the role of cytoplasmic peroxiredoxin‐2 (PRDX2), transforming growth factor‐beta‐induced protein ig‐h3 and NRF2.^70^ Their analysis pointed out that the expression of NRF2 indicates that aberrant NRF2‐mediated oxidative stress response (OSR) is a prominent feature of cervical carcinogenesis.^70^ Another fascinating study showed that cervical CCLs express markers associated with immunosurveillance. These were as follows: (a) MICA/B and CD95 (involved in tumour cell recognition); (b) CD39, CD73, CTLA‐4 (immune system escape); and (c) NKp30, NKp46, NKG2A and KIR3DL1 (typical markers of NK cells like). The authors concluded that these molecules might allow the CCLs to mimic the immune system.^71^

**TABLE 7:** List of common cervical cancer cell lines

Cell line	Patient	HPV status	Mutations	Primary tissue	Lit
C33A	66Y	Negative	MSI‐high DT – 1.36 d pseudodiploid p53 +; pRB +	Cervical squamous cell carcinoma. Part of CCLE and COSMIC.	Auersperg in 1964^86^
OMC‐4 (Osaka Medical College‐4)	47Y	Negative	Unknown MSI status DT – 63 h	Cervical adenocarcinoma.	Yamada T et al in 1987^87^
CaSki	40Y	Pos (HPV16)	MSI stable no TP53 mutation beta subunit of human chorionic gonadotropin (hCG)	Human papillomavirus‐related cervical squamous cell carcinoma. Metastatic site: Small intestine. Part of CCLE and COSMIC.	Pattillo R.A in 1977^88^
SiHa	55Y	Pos (HPV16)	MSI stable DT – 2.6 d p53 +; pRB + hypertriploid CL	Cervical squamous cell. Part of CCLE and COSMIC.	Friedl F. et al in 1970^89^
HeLa	30Y6M	Pos (HPV18)	MSI stable DT – 1.3‐2 d Four marker chromosomes P53 low, pRB normal	Endocervical adenocarcinoma. Part of CCLE and COSMIC.	Gey GO et al in 1952^67^
TMCC‐1	Age unspecified	Pos (HPV18)	Unknown MSI status DT – 53 h	Endocervical adenocarcinoma. Metastatic site: Pleural effusion.	Sakamoto M. et al in 1987^90^
ME180	66Y	Pos (HPV68)	MSI stable DT – 1.5 d P53 neg/pos, pRB + Heterozygous point mutation of PIK3CA p. Glu545Lys (c.1633G > A)	Cervical squamous cell carcinoma Metastatic site: Omentum. Part of CCLE and COSMIC	Sykes J. A. et al in 1970^91^
HT‐3	53‐58y^a^	Negative	MSI stable hypotriploid to hypertriploid DT – 2.48 d p53 +; pRB +	Metastatic site: Lymph node. Part of CCLE and COSMIC	Fogh J. and Trempe G. (1975) ^92^
C‐4‐I	41Y	Pos (HPV18)	MSI stable DT – 2 d	cervical squamous cell carcinoma Part of CCLE and COSMIC	Auersperg N in 1962^93^
C‐4‐II	41Y	Pos (HPV18)	MSI stable DT – 2.4 d	cervical squamous cell carcinoma	Auersperg N in 1962.^93^
MS751	47Y	Pos (HPV18) Pos (HPV45)	MSI stable DT – 2.4 d hypodiploid human cell line	epidermoid carcinoma Metastatic site: Lymph node Part of CCLE and COSMIC	Sykes J. A. et al in 1974
SW756	46y	Pos (HPV18)	MSI stable DT – 1.6 d Expressed genes: HLA A1, A24, B8, B44, Cw2, Cx, DR6Y; Le3; Le4; Le5	squamous cell carcinoma	Leibovitz A. in 1974

Note

Data summarized after the originators works as well as from the CELLOSAURUS databank.

Abbreviations: DT, doubling time; MSI, microsatellite.

^a^ Different indications – Indicated to be from a 58‐year‐old female patient on ATCC and from a 53‐year‐old on the Sloan Kettering tech transfer site.

## 6 CELL LINE CULTURING

**FIGURE 5:**
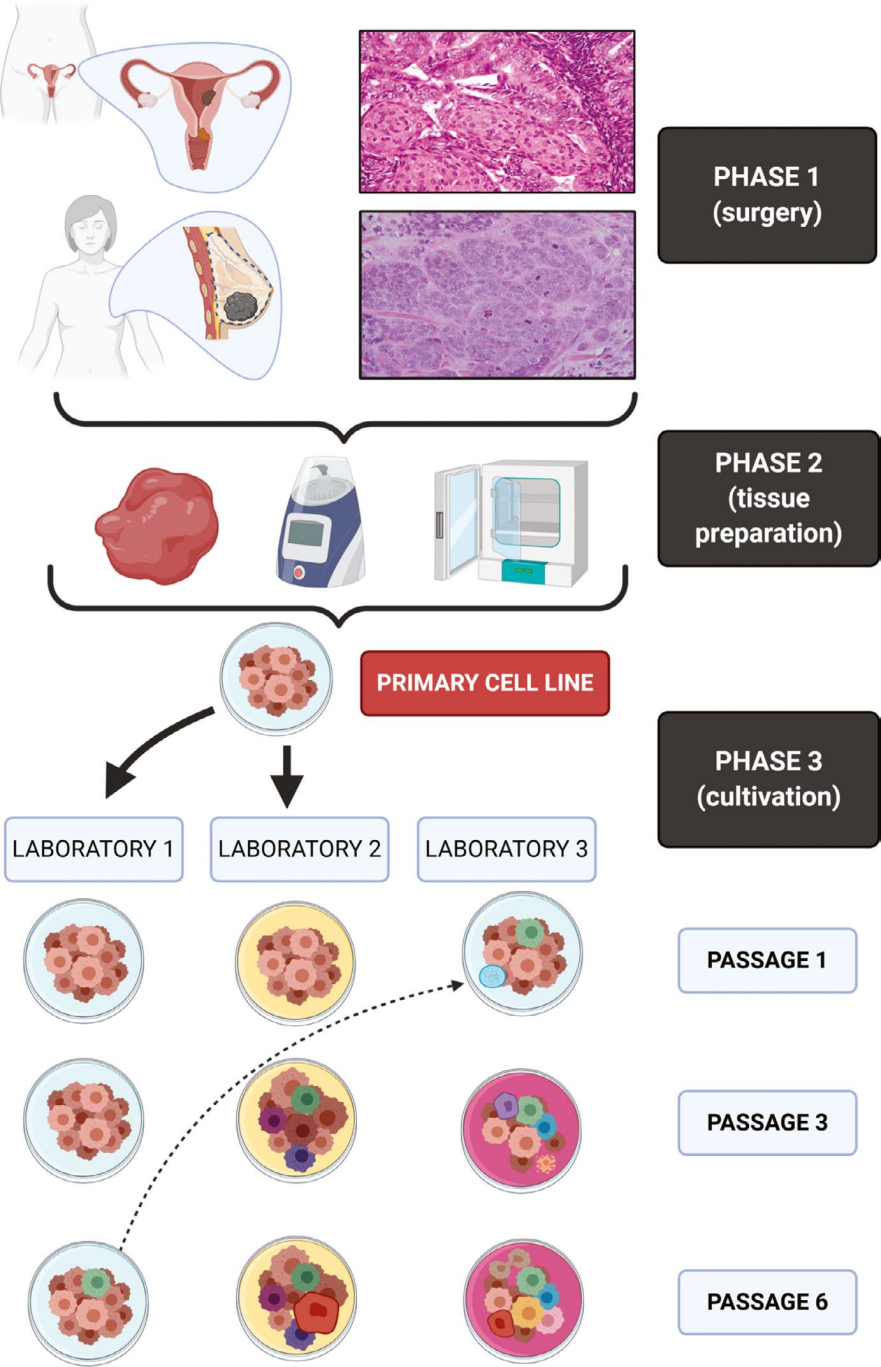
Cell line isolation and changes during cultivation. Phases 1 and 2 encompass the logistics of tissue retrieval and pre‐cultivation procedures (eg homogenization). Phase 3 shows the heterogeneity and susceptibility of CLs to mutate due to various changes. Laboratory 1 and laboratory 2 received commercially bought CLs. The CL from laboratory 1 mutated somewhere between passage 3 and 6. Laboratory 2 used from the beginning a different medium, which led to a variety of genotypical and phenotypical changes. Laboratory 3 borrowed a CL sample (already mutated) from laboratory 1. Due to careless handling, the CL got infected with mycoplasma. The addition of an antibiotic agent again led to a series of genotypical and phenotypical changes in the CL. Source: Histological images were used under the CC license from Wikimedia Commons (https://commons.wikimedia.org/wiki/File:Invasive_Ductal_Carcinoma_40x.jpg; https://commons.wikimedia.org/wiki/File:Endometrioid_endometrial_adenocarcinoma_very_high_mag.jpg). The figure itself was created with BioRender.com

The principle of cell culturing was established by Wilhelm Roux. He was an embryologist, who used warm saline to maintain chicken embryos for several days, thereby coming up with the tissue culture principle in 1885.^72^ The culturing process is a crucial step, and every mistake in its procedure can lead to a failed experiment (hence cease of cell growth). Changes in the environment can, via alterations in differentiation and gene expression signalling cascades drastically impact the cell morphology, intercellular interactions and cell polarity. Moreover, CCLs mostly stem from invasive high‐grade cancers, which means that genetic changes can occur much more often. These new mutations (de novo) may lead to phenotype changes. Furthermore, even perhaps seemingly small changes in the cell environmental such as changing the growth medium or temperature, different methods of cell culturing (xenografts, the addition of growth factors, 2D or 3D, etc) can lead to epigenetic alterations that affect the expression of genes.^73^ Through the years, a number of errors, contaminations and finally also reports with questionable reproducibility, consistency and validity have been exposed.^50^ These findings can have, in certain cases, little or, in other instances, dire consequences on the work and results of many. Ben‐David et al recently described the heterogenous nature of commonly used CCLs. The described differences extend from genetic information to phenotype and are proof of the instability and mutational potential of CLs (Figure 5). Consequently, the authors again stressed the need for a system of rules and measures which must be strictly enforced and followed. This system has been published in the international guidelines for the use of CLs in biomedical research. An important suggestion is determining the authenticity of the CL, which can be done via short tandem repeat (STR) profiling. The mechanism is the identification of variants in tetranucleotide microsatellite loci on multiple human chromosomes and comparing those to established databases. The same principle is used in paternity tests or other medico‐legal affairs. As an example: when applying this method in specific time or culturing intervals (eg first, third, fifth passage), the researcher is able to tell when the culture has undergone mutation (different STR profile). If we were all to follow the recommendations, then a lot of the problems would be solved.^74^ A succinct review on this topic has been given by Hynds and colleagues.^7^ An important point that the authors proposed is the reassessment of the ‘genomic landscape’, which undoubtedly changed a lot during the last years.^7^ Some measures for better consistency and reproducibility in cell culturing can be seen in Table 8.

**TABLE 8:** Measures for better consistency and reproducibility

Measures	Explanation	LIT
CL identification	To avoid misidentification, acquired CLs should come from a reliable source and must be authenticated, bought from a reliable source and banked for future use. Additional STR profiling is also important.	^94^
Mycoplasma testing	To avoid contamination good tissue culture practice and frequent testing should be performed to ensure that CLs are clear of contamination.	^95^
Use of validated reagents	To avoid a variety of errors only reagents of certified laboratory purity should be used. Decontamination should not be avoided but rather the experiment repeated.	^96^
Statistical standards	To avoid misinterpretations and promote transparency, mandatory reporting checklist that catalogued details of statistical information, experimental design and reagents, should be included.	.^7^, ^97^, ^98^, ^99^
Profiling	To avoid contaminations with other CLs and possible erroneous results, laboratory's own CLs should be compared to reference CL genomes.	^74^
Cryopreservation	To avoid loss of data and ensure replicability, preservation of the primary cultures and early passages with subsequent final comparison and validation of key findings before publication is of grave importance.	^74^
Reporting DTs	To promote transparency and replicability, accurate and diligent monitoring as well as reporting of DTs as well as a finite usage number of passages should be standard practice.	^74^
Standardized conditions	To ensure interlaboratory replicability, international standardized culture conditions for individual CLs and documentation of the heterogeneity metrics in datasheets should be standard practice.	^74^
Naming	To ensure coherent scientific reporting, the international naming guidelines should be used.	^100^

Abbreviations: CLs, cell lines; DT, doubling time; STR, short tandem repeat.

### 6.1 Author experience and recommendations

**FIGURE 6:**
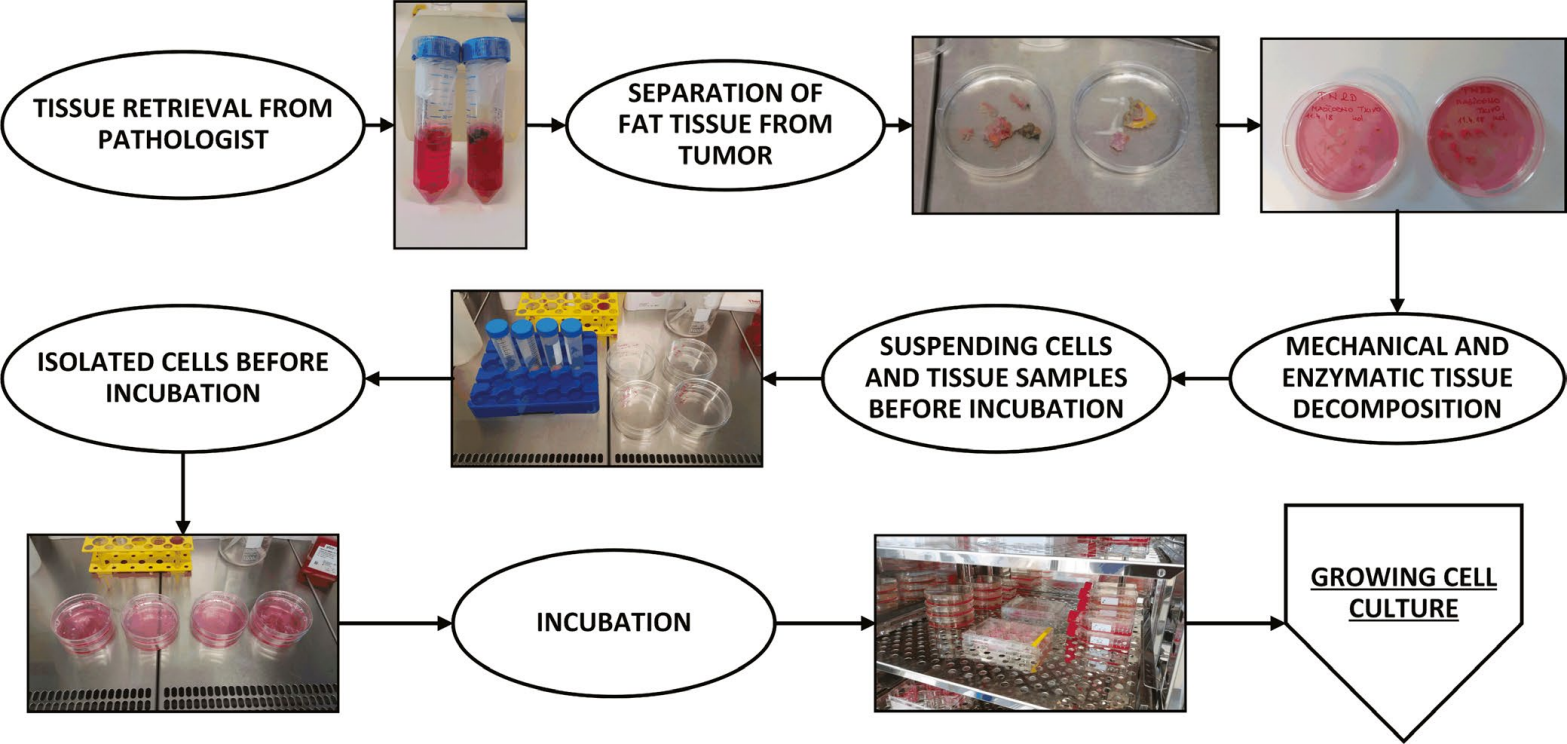
Cell culturing protocol

Culturing procedures have drastically changed over the years, especially with the development of new reagents and new materials. If comparing our current procedures with the past, we can now appreciate a much more streamlined process.^2^ The core principle, which is the usage of a culturing medium that supports the growth of cells as well as additions in the form of antibiotics that stop the growth of bacteria, is of course universally the same. However, the current procedures are much more uniform and perhaps easier to use as those described by the CL ‘pioneers’ (eg Dawe et al, AN3‐CA). Nevertheless, the protocols can still differ between laboratories. To illustrate, we present a simple workflow protocol (Figure 6) that we used for the isolation of our cell cultures and our characterization.^3^, ^75^, ^76^ Based on this workflow, we successfully cultured a TNBC CL. The naming was done in accordance with the current international guidelines (eg MFUM‐BrTNBC‐1).^3^, ^77^ Some of our other CLs include a human intestinal epithelial CL (HUIEC)^75^ as well as human chondrocytes.^76^

## 7 CONCLUSIONS

We presented different types of gynaecological cancers and their CLs as well as discussed aspects of their culturing. There is a growing body of evidence that, despite certain drawbacks, variations within CCLs can also be useful in regard to a more diverse genomic landscape. With more complex characterization methods, researchers would be able to expand their databases, investigate and research new interactions between CCLs as well as possibly discover new genotype/phenotype associations.^6^, ^7^ A good example is the study published in Nature about the results and genetic data of more than a 1000 CLs (eg NA splicing, DNA methylation, microRNA expression)^11^ and the recently established Models in Translational Oncology (MiTO) database, which will also help in the exchange of information on pre‐clinical model data relevant in translational cancer research.^1^ CLs remain an important and powerful tool for cancer research, but the genetic changes lead to variation across CL strains.^6^, ^7^ The adherence to the previously discussed rulesets, and the international guidelines help in minimizing replication failure between institutions. Nevertheless, the already present body of studies that report ‘false’ CLs remains. The authors Horbach and Halffman, therefore, proposed that notifications should be posted alongside previously published articles using misidentified CLs (eg ‘expressions of concern’). This would not be a ‘witch hunt’ (eg retraction hunt) but simply alert the reader that there may be an issue with a paper, when, as the authors nicely said, ‘the full story is not yet clear’. Furthermore, the authors recommended to increase the visibility of utilized CLs in articles by mentioning the employed CLs in easily searchable parts of their article (eg abstract and keywords).^68^

## CONFLICT OF INTEREST

The authors declare no conflict of interest. The funders had no role in the design of the study; in the collection, analyses or interpretation of data; in the writing of the manuscript; or in the decision to publish the results.

## AUTHOR CONTRIBUTIONS

**Kristijan Skok:** Conceptualization (lead); Formal analysis (equal); Investigation (equal); Methodology (equal); Visualization (equal); Writing‐original draft (lead); Writing‐review & editing (equal). **Lidija Gradišnik:** Conceptualization (equal); Formal analysis (equal); Investigation (equal); Writing‐review & editing (equal). **Uroš Maver:** Conceptualization (equal); Formal analysis (equal); Investigation (equal); Methodology (equal); Supervision (equal); Writing‐original draft (equal); Writing‐review & editing (equal). **Nejc Kozar:** Writing‐review & editing (equal). **Monika Sobočan:** Writing‐review & editing (equal). **Iztok Takač:** Formal analysis (equal); Investigation (equal); Writing‐review & editing (equal). **Darja Arko:** Formal analysis (equal); Investigation (equal); Writing‐review & editing (equal). **Rajko Kavalar:** Conceptualization (equal); Formal analysis (equal); Investigation (equal); Supervision (equal); Writing‐original draft (equal); Writing‐review & editing (equal).
